# Codebook for rating clinical communication skills based on the Calgary-Cambridge Guide

**DOI:** 10.1186/s12909-020-02050-3

**Published:** 2020-05-06

**Authors:** Else Dalsgaard Iversen, Maiken Overbeck Wolderslund, Poul-Erik Kofoed, Pål Gulbrandsen, Helle Poulsen, Søren Cold, Jette Ammentorp

**Affiliations:** 1 Health Services Research Unit, Lillebaelt Hospital, Beriderbakken 4, DK-7100 Vejle, Denmark; 2 Institute for Regional Health Research, University of Southern Denmark, Winsløwparken 19, Odense, Denmark; 3 OPEN, Odense Patient data Explorative Network, J. B. Winsløws Vej 9a, 5000 Odense, Denmark; 4 Department of Paediatrics, Lillebaelt Hospital, Sygehusvej 24, DK-6000 Kolding, Denmark; 5 Institute of Clinical Medicine, Campus Ahus, University of Oslo, 1478 Nordbyhagen, Oslo, Norway; 6 HØKH, Akershus University Hospital, Sykehusveien 25, 1478 Nordbyhagen, Norway; 7 Department of Gastrointestinal Surgery, Lillebaelt Hospital, Sygehusvej 24, DK-6000 Kolding, Denmark; 8 Department of Oncology, Odense University Hospital, J. B. Winsløws Vej 4, DK-5000 Odense, Denmark

**Keywords:** Assessment tool, Communication skills training, Audio recordings, Calgary-Cambridge guide, Interrater reliability, Codebook, Observation Scheme-12

## Abstract

**Background:**

The aim of the study was to confirm the validity and reliability of the Observation Scheme-12, a measurement tool for rating clinical communication skills.

**Methods:**

The study is a sub-study of an intervention study using audio recordings to assess the outcome of communication skills training. This paper describes the methods used to validate the assessment tool Observation Scheme-12 by operationalizing the crude 5-point scale into specific elements described in a codebook. Reliability was tested by calculating the intraclass correlation coefficients for interrater and intrarater reliability.

**Results:**

The validation of the Observation Scheme-12 produced a rating tool with 12 items. Each item has 0 to 5 described micro-skills. For each item, the codebook described the criteria for delivering a rating from 0 to 4 depending on how successful the different micro-skills (or number of used jargon words) was accomplished. Testing reliability for the overall score intraclass correlation coefficients was 0.74 for interrater reliability and 0.86 for intrarater reliability. An intraclass correlation coefficient greater than 0.5 was observed for 10 of 12 items.

**Conclusion:**

The development of a codebook as a supplement to the assessment tool Observation Scheme-12 enables an objective rating of audiotaped clinical communication with acceptable reliability. The Observation Scheme-12 can be used to assess communication skills based on the Calgary-Cambridge Guide.

## Background

Effective and competent clinical communication skills are widely acknowledged as a key component of high-quality healthcare, and have a positive impact on health outcomes [1, 2], including better adherence to treatment [3]. In contrast, communication breakdown, particularly verbal communication breakdown [4], can lead to malpractice claims and complaints in hospital care [5]. Communication skills training for health care providers (HCPs) is recommended for promoting good communication in health care, and methods have been developed for teaching and training purposes [6, 7].

The Calgary-Cambridge Guide (C-CG) is a well-known approach to teaching and training clinical communication skills. It was introduced by Kurtz and Silverman in 1996 [8] to define the communication curriculum and to develop a feasible teaching method. Currently, it is used worldwide and was last updated with a third edition in 2013 [9]. The C-CG was not intended to be an assessment tool. However, during teaching sessions, it has been used as a guide to assess the specific communication skills performed and to provide systematic and structured feedback.

With the introduction of teaching programmes, many assessment tools have been developed [10–12], including tools based on the C-CG [13–22]. The tools differ in the number of items, response scales, settings, and aims of the assessment. One tool used three items, as the aim of the study was to assess agenda making [17]. Another tool excluded items measuring the beginning and closing of the consultations [16]. The most common use of the C-CG as an assessment tool is to evaluate communication throughout consultation [13, 14, 18, 20]. Some tools have been developed for an Objective Structured Clinical Examination (OSCE) [15, 19], while others have been developed for rating audio or video recordings of the consultation [13, 22]. The tools have been used in different countries [14, 17, 21].

In Denmark, an assessment tool based on the C-CG was developed by two of the co-authors (JA and PK) [20] with the purpose of comparing medical students’ self-efficacy in communication skills to the observed ratings using simulated patients and an examiner during an OSCE [20]. The questionnaire was a useful and reliable tool for measuring communication skills based on the C-CG. As the questionnaire was familiar to the authors and tested in a Danish setting, we decided to confirm the validity and reliability before using it in an intervention study where audio recordings were planned to be rated in a pre and post design. The questionnaire was named Observation Scheme – 12 (OS-12).

The aim of the study was to confirm the validity and reliability of Observation Scheme-12, a measurement tool for rating clinical communication skills.

## Methods

### Setting

The study was part of an intervention study investigating the impact of the implementation of communication skills training based on C-CG at a large regional hospital in Denmark (“Clear Cut Communication with Patient”) [23]. The consultations occurred at the interdisciplinary outpatient clinic at the Spine Centre of Southern Denmark, Lillebaelt Hospital.

### Study sample

During the period from 2014 to 2015, 51 HCPs were asked to audio record 10 encounters before and after participating in the communication skills training. All audio recordings documented individual consultations between patients presenting with back or neck pain and a medical doctor, nurse, physiotherapist or chiropractor. Patients were informed about the purpose of the study at the beginning of the consultation and asked whether they wanted to participate. The HCPs turned on the audio recorder after the patients had provided informed consent.

### Assessment tool

The OS-12 contains 12 items covering the following six domains: initiating the session, gathering information, building the relationship, explanation and planning, providing structure, and closing the session. Each item was rated on a 5-point scale with the following levels of quality: 0 – ‘Poor’, 1 – ‘Fair’, 2 – ‘Good’, 3 – ‘Very good’, and 4 – ‘Excellent’. Consequently, the overall score ranged from 0 to 48 points.

### Content validation

A panel of four researchers and three teachers were selected to judge the ability of the OS-12 to measure the construct of the provided communication skills training. The researchers had been a part of developing the communication skills training program, “Clear Cut Communication with Patient”, based on the C-CG and the teachers were trained as communication trainers in the program.

### Codebook development

The codebook was developed by rating 23 audio recordings from seven HCPs (Table 1 describes the characteristics of the included patients and HCP’s). The codebook described how points should be allocated in terms of distinguishing between similar scores. The coders divided the micro-skills from each item into four groups to systematize and quantify the points to be allocated. As the full length of some consultations had not been recorded, the option of rating an item as “not applicable” was added.

**Table 1:** Characteristics of patients and HCP’s participating in codebook development and interrater reliability (IRR) evaluation

	Codebook development N (%)	Evaluation of the IRR N (%)
Patients	23	83
HCPs	7	30
Audios per HCP, *mean* (range)	3.3 (1–5)	2.8 (1–3)
Gender, female		
Patients	12 (52%)	48 (57%)
HCPs	6 (86%)	24 (80%)
Age in years		
Patients, *mean* (range)	48 (25–79)	47 (17–84)
Profession – HCPs		
Physiotherapist	5 (71%)	15 (50%)
Chiropractor	0 (0%)	7 (23%)
Nurse	2 (29%)	6 (20%)
Doctor	0 (0%)	2 (7%)
Duration of the encounter, min		
*mean* (range)	21.3 (4–42)	20.9 (6–42)

### Coding procedure

Two of the authors (EI and HP) coded the recordings. These authors are an experienced medical doctor and an experienced nurse, respectively. The nurse had completed the same communication skills training programme as the participating HCPs and the medical doctor had experience in teaching communication skills to medical students.

The coders listened to the audio recordings while making notes on a handwritten form of the OS-12 before transferring the results into a SurveyXact solution, an online data management system. The coders found no need for transcriptions of the audio recordings as they manually wrote important sentences and described how micro-skills were demonstrated to support the points given.

### Outcome measures and statistical analysis

The OS-12 is intended to measure communication throughout the consultation, and therefore our primary measurement of reliability was the overall score calculated by adding the scores for the 12 items. Reliability was assessed by calculating the intraclass correlation coefficient (ICC) [24]. It is based on two-way random-effect with an absolute agreement for interrater reliability [25]. The ICC for intrarater reliability was also based on the two-way model, but with a mixed-effect [25]. The ICC for each item was calculated to investigate whether some items had a lower correlation than others. The statistical analysis was conducted using the STATA/IC 15.0 software package.

## Results

Audio recordings from 30 HCPs were included. See Table 1 for the characteristics.

### Content validation

The panel of researchers and teachers determined that every item was relevant and matched the communications skills training based on the C-CG. In addition, they suggested adding micro-skills from the C-CG to increase the understanding of the items. The micro-skills selection was based on the teacher’s experience from the first training courses and were included if both researchers and teachers agreed that the micro-skills were essential to the item. For some items, it was decided to merge two micro-skills from the C-CG as they were considered to be connected. In item 1, “Identifies problems the patient wishes to address” the micro-skills “making an opening question” was merged with “listening actively” as the panel decided that HCPs had to give space for the patient to answer if they used an opening question. In addition, the panel found it difficult to negotiate an agenda without screening for further issues. Therefore those two micro-skills were merged. The results from the content validation are shown in Table 2.

**Table 2:** Codebook with criteria for points allocated to each item

**Domains**	**Item 1: Identifies problems the patient wishes to address**		
**Initiating the session**	**Micro-skills**	**Demonstrated micro-skills /criteria**	**Points**
	1. Greets patients 2. Introduces oneself, one’s role and the nature of the interview 3. Demonstrates respect and interest; attends to patient’s physical comfort 4. Uses an appropriate opening question/listens attentively 5. Confirms issues to be discussed/screens for further questions and negotiates the agenda	Micro-skill 1 and micro-skill 2	One
		Micro-skill 1 and micro-skill 2 together with one of the other micro-skills.	Two
		Micro-skill 1 and micro-skill 2 together with two of the other micro-skills.	Three
		All micro-skills are demonstrated	Four
	**Item 2: Clarifies the patient’s prior knowledge and desire for information**		
**Gathering information**	**Micro-skills**	**Demonstrated micro-skills /criteria**	**Points**
	1. Listens attentively, allowing the patient to complete statements without interruption and leaving space for patient 2. Encourages the patient to tell the story of the problem(s) from when it/they first started to the present in his/her own words 3. Uses open and closed questioning techniques, appropriately moving from open to closed questions 4. Clarifies patient’s statements that are unclear or need amplification 5. Periodically summarizes, invites the patient to correct the interpretation or provide further information	One micro-skill	One
		Two or three micro-skills	Two
		Four micro-skills	Three
		All micro-skills are demonstrated	Four
	**Item 3: Uses easily understood language, avoids jargon**		
**Building a relationship**	**Micro-skills**	**Demonstrated micro-skills /criteria**	**Points**
	No micro-skills	Used ten or more medical words	One
		Used between four and nine medical words	Two
		Used two to three medical words	Three
		Used one or none medical words	Four
	**Item 4: Uses appropriate non-verbal behaviour**		
	**Micro-skills**	**Demonstrated micro-skills /criteria**	**Points**
	1. Calm speaking paces 2. No interruptions 3. Leaves space for the patient to talk 4. Pausing	One micro-skill	One
		Two micro-skills	Two
		Three micro-skills	Three
		All micro-skills are demonstrated	Four
	**Item 5: Provide support: expresses concern and willingness to help**		
	**Micro-skills**	**Demonstrated micro-skills /criteria**	**Points**
	1. Accepts the legitimacy of the patient’s views and feelings; is not judgmental 2. Uses empathy to communicate understanding and appreciation of the patient’s feelings 3. Provides support: expresses concern, understanding, and willingness to help.	Some support provided, but no micro-skill is demonstrated	One
		One micro-skill	Two
		Two micro-skills	Three
		All micro-skills are demonstrated	Four
	**Item 6: Structures the interview in a logical sequence**		
**Providing structure**	**Micro-skills**	**Demonstrated micro-skills /criteria**	**Points**
	Progresses from one section to another using 1. Signposting 2. Transitional statements 3. Rationale for the next section	Some structure is present, but no micro-skill is demonstrated	One
		One micro-skill	Two
		Two micro-skills	Three
		All micro-skills are demonstrated	Four
	**Item 7: Attends to timekeeping, and keeps the interview on track**		
	**Micro-skills**	**Demonstrated micro-skills /criteria**	**Points**
	1. Structures the interview **based on** the C-CG 2. Attending to timing 3: Keeping the interview on track	Some structure is present, but no micro-skill is demonstrated	One
		One micro-skill	Two
		Two micro-skills	Three
		All micro-skills are demonstrated	Four
	**Item 8: Shares thoughts and reflections with the patient**		
**Explanation and planning**	**Micro-skills**	**Demonstrated micro-skills /criteria**	**Points**
	1. Assesses patient’s starting point (preferably using tailored explanations and illustrations) 2. Provides information in manageable chunks, assesses understanding uses patient’s responses as a guide for the best way to proceed 3. Providing the correct amount and type of information to individual patients	Some thoughts/reflections are provided, but no micro-skill is demonstrated	One
		One micro-skill	Two
		Two micro-skills	Three
		All micro-skills are demonstrated	Four
	**Item 9: Checks the patient’s understanding**		
	**Micro-skills**	**Demonstrated micro-skills /criteria**	**Points**
	1. Organizes the explanation (uses summarizing) 2. Assesses the patient’s understanding (asks the patient to summarize the information he/she was provided) 3. Asks the patient what other information would be helpful, addresses patient’s needs for information	A question was asked, but no micro-skill is demonstrated	One
		One micro-skill	Two
		Two micro-skills	Three
		All micro-skills are demonstrated	Four
	**Item 10: Negotiates a mutual plan of action**		
	**Micro-skills**	**Demonstrated micro-skills /criteria**	**Points**
	1. Explores options with the patient 2. Involves the patient in decision making 3. Negotiates a mutually acceptable plan	Some negotiation occurs, but no micro-skill is demonstrated	One
		One micro-skill	Two
		Two micro-skills	Three
		All micro-skills are demonstrated	Four
	**Item 11**: **Contracts with the patient about next steps**		
**Closing the session**	**Micro-skills**	**Demonstrated micro-skills /criteria**	**Points**
	1. Contracts with the patient about the next steps 2. Safety nets, e.g., phone number and other lifelines	One micro-skill is initiated but not completed	One
		One micro-skill	Two
		One micro-skill is completed and the other is initiated	Three
		All micro-skills are demonstrated	Four
	**Item 12: Summarizes the session briefly and clarifies the plan of care**		
	**Micro-skills**	**Demonstrated micro-skills / criteria**	**Points**
	1. Final confirmation of patient understanding 2. Summarizes the session briefly and clarifies the plan of care 3. Finally confirms that the patient agrees and is comfortable with the plan	Some type of summary is provided, but no micro-skill is demonstrated	One
		One micro-skill	Two
		Two micro-skills	Three
		All micro-skills are demonstrated	Four

Be very precise about coding the demonstrated skills in the domains in which they occurred

Zero points were recorded if an item was not apparent

If the audio recording stops before all information is provided, items 8, 9 and 10 were coded as “not applicable”

The structure was coded if the audio recording did not stop during “Initiating the session” and “Gathering information”

### Codebook development

Table 2 also presents the codebook with an overview of the criteria for points allocated to each item of the OS-12. It is based on an assessment of the demonstrated micro-skills and other types of behaviours as they appeared in the audio recordings. Before using the OS-12 and the codebook, an understanding of the micro-skills as described in the C-CG [9] is necessary, as the coding procedure is based on the raters’ abilities to identify these micro-skills.

Four items were more troublesome for the coders to describe than others. Therefore, details regarding the coding of these items are provided below.

Item 3, “Uses easily understood language, avoids jargon”, does not contain any micro-skills. Consequently, the coders decided to allocate points according to the number of medical terms used. However, an issue was that some words were clearly medical jargon, for example: “cerebrum”, “column” or the question “how is your general condition?” whereas other words were more difficult to specify as medical jargon, such as, “prognosis”, “paracetamol” and a very commonly used word, “functioning”. The coders concluded that the use of medical jargon was acceptable as long as the words were explained to the patient. For difficult words, the coders were required to judge whether the patient understood the words based on subsequent expressions in the consultation. If the patients did not understand the word, it was coded as medical jargon.

Item 4, “Uses appropriate non-verbal behaviour”, was challenging to rate in audio recordings instead of videos. The distinction listed below was made between the four micro-skills. The tone of voice of the HCP was used to assess a “calm speaking pace”, whereas “pausing” meant that the HCP allowed silence during the conversation. Points for “no interruptions” were given when the HCP listened to the patients without interruptions nor finishing the patient’s sentences. Finally, “Leaves space for the patient to talk” was present when the HCP allowed patients to tell their stories and enabled the patients to talk about their worries and concerns.

In item 7, “Attends to timekeeping, and keeps the interview on track”, the coders listened for the ability of the HCP to structure the consultation according to the 4 C-CG domains: initiating the session, gathering information, explanation, and planning and closing the session. When the HCP demonstrated proficiency in these four domains they received two points. Thus, if the coders disagreed on whether the HCP convincingly demonstrated the four domains, they also disagreed on item 7.

Coding item 9 “Checks the patient’s understanding” proved to be difficult, as the micro-skills were rarely demonstrated. The use of a summary, an essential part of the first micro-skill, was occasionally performed by the HCP, but very few HCPs had the patients summarize the information or confirmed that the patient had understood the information provided to them. The last micro-skill, “Asks patients what other information would be helpful, address patient’s needs for information”, was often demonstrated at the end of the consultation and was sometimes difficult to differentiate from the micro-skill: “Finally checks that the patient agrees and is comfortable with the plan” from item 12, as some HCP asked “are there any uncertainties?” or “anything else we need to talk about?” when closing the consultation. Consequently, it was specified in the codebook to give points only if the demonstrated micro-skill occurred in the right domain.

### Interrater reliability

The main outcome measurement for the ICC was the overall score, and the codebook resulted in good interrater reliability (IRR), as the ICC was 0.74 (95% CI 0.52–0.85), Table 3. The ICC was greater than 0.5 for 10 items, while the ICCs for two items, “Attends to timekeeping, and keeps the interview on track” and “Checks patient’s understanding”, were below this threshold. Items 1 and 2 were rated in 82 of 83 cases, as the audio recorder was not turned on at the beginning of the consultation on one occasion. Items 11 and 12 were rated in 80 of 83 cases as the audio recorder stopped in three cases before the closing of the consultations.

**Table 3:** Intraclass correlation coefficients (ICCs) for interrater and intrarater reliability

Item		Interrater reliability *N* = 83		Intrarater reliability *N* = 20	
		ICC	95% Cl	ICC	95% Cl
1	Identifies problems the patient wishes to address	0.74	0.60–0.83	0.55	−0.14-0.82
2	Clarifies the patient’s prior knowledge and desire for information	0.68	0.48–0.80	0.35	−0.64-0.72
3	Uses easily understood language, avoids jargon	0.55	0.31–0.71	0.75	0.39–0.90
4	Uses appropriated non-verbal behaviour	0.71	0.55–0.81	0.75	0.38–0.90
5	Provides support: expresses concern and willingness to help	0.59	0.11–0.78	0.78	0.45–0.91
6	Structures the interview in logical sequence	0.56	0.33–0.72	0.39	−0.54-0.75
7	Attends to time keeping, and keeps the interview on track	0.29	−0.11-0.54	0.76	0.39–0.90
8	Shares thoughts and reflections with the patient	0.51	0.23–0.69	0.43	−0.4-0.77
9	Checks the patient’s understanding	0.15	−0.32-0.45	0.76	0.40–0.91
10	Negotiates a mutual plan of action	0.74	0.60–0.83	0.78	0.43–0.91
11	Contracts with the patient about the next steps	0.88	0.80–0.93	0.91	0.76–0.96
12	Summarizes the session briefly and clarifies the plan of care	0.65	0.46–0.77	0.43	0.44–0.77
	Overall score	0.74	0.52–0.85	0.86	0.65–0.94

### Intrarater reliability

With an interval of 3 months, one of the authors (EI) re-rated 20 audio recordings. The ratings correlated with the overall score, with an ICC of 0.86 (95% CI 0.64–0.94).

## Discussion

In this study, we present the validation and the process of developing a codebook to establish reliability in rating clinical communication skills using the OS-12 assessment tool. Based on guidelines [26], good interrater reliability (0.74) and excellent intrarater reliability (0.86) were observed for the overall score when the codebook was used alongside the OS-12 assessment tool.

Only a few other studies have reported the IRR when using assessment tools based on the C-CG. Simmenroth-Nayda et al. (2012) reported Pearson’s r correlation coefficient of 0.62 for the overall score in 2012 [21]. In 2014 [27], the same group reported poor-fair reliability (ICC ranging from 0.05–0.57) on individual items from the C-CG. Thus, coding communication is difficult and despite the codebook, we were not able to observe a sufficient ICC (> 0.4) [26] for item 7 “Attends to timekeeping and keeps the interview on track” and item 9 “Checks patient’s understanding”.

The two coders allocated two points for item 7 “Attends to timekeeping, and keeps the interview on track” if the interview was structured based on the C-CG, including initiating the session, gathering information, explanation and planning, and closing the session. However, if the coders disagreed on the successful fulfilment of other items, such as item 2 “Clarifies the patient’s prior knowledge and desire for information” or item 12 “Summarizes the session briefly and clarifies the plan of care”, they also disagreed on item 7, making item 7 sensitive to disagreement on other items (data not shown). When the coders talked about item 9, they defined the meaning of “checking for patient’s understanding” and the micro-skills related to this item. They concluded that the HCPs must confirm that the patient understood the information provided in the consultation. However, because the raters did not have access to the patients’ non-verbal responses, they were unable to easily assess whether the patients understood the information. HCPs may have accepted a nod as an acknowledgement that the patient understood the explanation. Only a few HCPs explicitly asked patients to repeat or summarize the information provided. Generally, HCPs asked a simple closing question, e.g., “Do you understand?” or “Do you have any questions?”, and accepted a yes or a no, respectively, as verification of the patient’s understanding, making the judgement of whether the patient actually understood the information difficult. The confirmation of a patient’s understanding is a well-known challenge, as HCPs have been shown to overestimate and rarely thoroughly confirm the patient’s understanding [28]. Likewise, patients overestimate what they understand or do not express their lack of understanding [29].

The difficulties with an insufficient ICC for items 7 and 9 indicate the well-known problem of a low ICC when items have low scores or variance, as minor disagreements subsequently have a greater impact on the IRR [24, 30]. However, this problem was not observed in the present study, and a valuable discussion is whether items with a low ICC should be excluded. Nevertheless, the OS-12 is based on the C-CG and therefore builds on the assumption that every item is essential and relevant to the consultation. Consequently, no items were excluded and we recommend using the “not applicable” response option only due to technical difficulties or similar situations. In this study, none of the items were coded “not applicable” if the entire encounter was recorded.

We used a 5-point scale in the codebook because it was tested in the original study [20]. Other researchers have used two-point [17, 31], three-point [14, 19], four-point [13, 18] or five-point scales [27] when rating communication skills based on the C-CG. We recommend maintaining the 5-point scale when utilizing the OS-12, as all micro-skills are divided into groups of five.

The two coders had similar characteristics (e.g., training, experience, and gender) and previous experience in coding [32]. However, they had different professional backgrounds (e.g., a nurse and a doctor). According to other studies [33], coders with the same gender, professional background, and coding experience generate a higher IRR. In the present study, a decision was made to have coders from different professional disciplines rate the audio recordings, because the recordings were obtained from an interdisciplinary clinic with different HCPs represented.

The fact that the encounters were audio-recorded instead of video recorded was a limitation of the study resulting in an incomplete rating of the non-verbal communication. Without access to visual documentation of the encounter, it was impossible to assess how the body language and the interaction between the HCP and the patient affected the relationship. However, in order to be able to assess parts of the non-verbal communication, we chose to rate calm non-speaking paces, no interruptions of the patient, leaving space for the patient to talk and pausing. The audio solution was chosen because it was the most feasible method in that setting. A second limitation was that the OS-12 did not include every micro-skills from the C-CG. The C-CG contains 73 different micro-skills [9] and in this study, the expert group selected the ones that were given the highest priority at the training course. Consequently, the OS-12 reflects the selected skills and the coding tool has to be used considering this limitation. Furthermore, as the C-CG is a generic communication skill teaching strategy the OS-12 may be utilized to code these skills in other countries and settings where communication skills training is based on the C-CG. However, studies are required to investigate whether similar results can be obtained in other countries and when the OS-12 is applied in other settings and countries validation is recommended including careful consideration of which micro-skills have been given priority in the specific training course.

## Conclusions

The utilization of a codebook as a supplement to the OS-12 assessment tool fosters an objective rating of clinical communication skills. It provides acceptable interrater and intrarater reliabilities for the overall score when audio recordings are coded separately by two raters. The OS-12 can be used to assess the communication skills of HCPs and evaluate communication throughout the HCP-patient encounter. The OS-12 is particularly recommended as an assessment tool if communication is based on the Calgary-Cambridge Guide.

## Abbreviations

OS-12: Observation Scheme-12
HCP: Health Care Provider
C-CG: Calgary-Cambridge Guide
OSCE: Objective Structured Clinical Examination
ICC: Intraclass Correlation Coefficient
IRR: Interrater reliability

## References

[1] Street RL, Makoul G, Arora NK, Epstein RM (2009). How does communication heal? Pathways linking clinician-patient communication to health outcomes. Patient Educ Couns.

[2] Georgopoulou S, Prothero L, D’Cruz DP (2018). Physician-patient communication in rheumatology: a systematic review. Rheumatol Int.

[3] Zolnierek KB, Dimatteo MR (2009). Physician communication and patient adherence to treatment: a meta-analysis. Med Care.

[4] Greenberg CC, Regenbogen SE, Studdert DM, Lipsitz SR, Rogers SO, Zinner MJ, Gawande AA (2007). Patterns of communication breakdowns resulting in injury to surgical patients. J Am Coll Surg.

[5] Ferguson B, Geralds J, Petrey J, Huecker M (2018). Malpractice in emergency medicine-a review of risk and mitigation practices for the emergency medicine provider. J Emerg Med.

[6] Berkhof M, van Rijssen HJ, Schellart AJ, Anema JR, van der Beek AJ (2011). Effective training strategies for teaching communication skills to physicians: an overview of systematic reviews. Patient Educ Couns.

[7] Dwamena F, Holmes-Rovner M, Gaulden CM, Jorgenson S, Sadigh G, Sikorskii A, Lewin S, Smith RC, Coffey J, Olomu A (2012). Interventions for providers to promote a patient-centred approach in clinical consultations. Cochrane Database Syst Rev.

[8] Kurtz SM, Silverman JD (1996). The Calgary-Cambridge referenced observation guides: an aid to defining the curriculum and organizing the teaching in communication training programmes. Med Educ.

[9] Kurtz S, Silverman J, Draper J (2013). Skills for communication with patients.

[10] Zill JM, Christalle E, Muller E, Harter M, Dirmaier J, Scholl I (2014). Measurement of physician-patient communication--a systematic review. PLoS One.

[11] Gillis AE, Morris MC, Ridgway PF (2015). Communication skills assessment in the final postgraduate years to established practice: a systematic review. Postgrad Med J.

[12] Schirmer JM, Mauksch L, Lang F, Marvel MK, Zoppi K, Epstein RM, Brock D, Pryzbylski M (2005). Assessing communication competence: a review of current tools. Fam Med.

[13] Skelly K, Rosenbaum M, Barlow P, Priebe G (2019). Comparing resident-patient encounters and case presentations in a family medicine clinic. Med Educ.

[14] Burt J, Abel G, Elmore N, Campbell J, Roland M, Benson J, Silverman J (2014). Assessing communication quality of consultations in primary care: initial reliability of the global consultation rating scale, based on the Calgary-Cambridge guide to the medical interview. BMJ Open.

[15] Berney A, Carrard V, Schmid Mast M, Bonvin R, Stiefel F, Bourquin C (2017). Individual training at the undergraduate level to promote competence in breaking bad news in oncology. Psychooncology.

[16] Nikendei C, Bosse HM, Hoffmann K, Moltner A, Hancke R, Conrad C, Huwendiek S, Hoffmann GF, Herzog W, Junger J, Schultz JH (2011). Outcome of parent-physician communication skills training for pediatric residents. Patient Educ Couns.

[17] Roh H, Park KH, Jeon YJ, Park SG, Lee J (2015). Medical students’ agenda-setting abilities during medical interviews. Korean J Med Educ.

[18] Sommer J, Lanier C, Perron NJ, Nendaz M, Clavet D, Audetat MC (2016). A teaching skills assessment tool inspired by the Calgary-Cambridge model and the patient-centered approach. Patient Educ Couns.

[19] Wild D, Nawaz H, Ullah S, Via C, Vance W, Petraro P (2018). Teaching residents to put patients first: creation and evaluation of a comprehensive curriculum in patient-centered communication. BMC Med Educ.

[20] Ammentorp J, Thomsen JL, Jarbol DE, Holst R, Ovrehus AL, Kofoed PE (2013). Comparison of the medical students’ perceived self-efficacy and the evaluation of the observers and patients. BMC Med Educ.

[21] Simmenroth-Nayda A, Weiss C, Fischer T, Himmel W (2012). Do communication training programs improve students’ communication skills?--a follow-up study. BMC Res Notes.

[22] Burt J, Abel G, Elmore N, Newbould J, Davey A, Llanwarne N, Maramba I, Paddison C, Benson J, Silverman J, Elliott MN, Campbell J, Roland M (2018). Rating communication in GP consultations: the association between ratings made by patients and trained clinical raters. Med Care Res Rev.

[23] Ammentorp J, Graugaard LT, Lau ME, Andersen TP, Waidtlow K, Kofoed PE (2014). Mandatory communication training of all employees with patient contact. Patient Educ Couns.

[24] Hallgren KA (2012). Computing inter-rater reliability for observational data: an overview and tutorial. Tutor Quant Methods Psychol.

[25] Koo TK, Li MY (2016). A guideline of selecting and reporting Intraclass correlation coefficients for reliability research. J Chiropr Med.

[26] Cicchetti D (1994). Guidelines, criteria, and rules of thumb for evaluating normed and standardized assessment instrument in psychology.

[27] Simmenroth-Nayda A, Heinemann S, Nolte C, Fischer T, Himmel W (2014). Psychometric properties of the Calgary Cambridge guides to assess communication skills of undergraduate medical students. Int J Med Educ.

[28] Horwitz LI, Moriarty JP, Chen C, Fogerty RL, Brewster UC, Kanade S, Ziaeian B, Jenq GY, Krumholz HM (2013). Quality of discharge practices and patient understanding at an academic medical center. JAMA Intern Med.

[29] Engel KG, Heisler M, Smith DM, Robinson CH, Forman JH, Ubel PA (2009). Patient comprehension of emergency department care and instructions: are patients aware of when they do not understand?. Ann Emerg Med.

[30] Fossli Jensen B, Gulbrandsen P, Benth JS, Dahl FA, Krupat E, Finset A (2010). Interrater reliability for the four habits coding scheme as part of a randomized controlled trial. Patient Educ Couns.

[31] Greenhill N, Anderson C, Avery A, Pilnick A (2011). Analysis of pharmacist-patient communication using the Calgary-Cambridge guide. Patient Educ Couns.

[32] Poulsen H, Iversen ED, Ammentorp J (2017). The development and initial validation of an observational tool for measuring patient participation in clinical consultations. Eur J Pers Cent Healthc.

[33] Mortsiefer A, Karger A, Rotthoff T, Raski B, Pentzek M (2017). Examiner characteristics and interrater reliability in a communication OSCE. Patient Educ Couns.

